# Pembrolizumab-Induced Eruptive Keratoacanthomas Managed With Intralesional Corticosteroids: A Case Report

**DOI:** 10.7759/cureus.108171

**Published:** 2026-05-03

**Authors:** Kevin Varghese, Develyn Vetos, Jason Woods, Jesalyn Tate, Ting Wang-Weinman

**Affiliations:** 1 Dermatology, University of Kansas Medical Center, Kansas City, USA

**Keywords:** dermatology and dermatologic surgery, immune-mediated adverse events, nonmelanoma skin cancer, pd-1 inhibitors, scc-squamous cell carcinoma

## Abstract

Pembrolizumab, a programmed cell death protein 1 (PD-1) inhibitor, is approved for multiple malignancies, including renal cell carcinoma in combination with axitinib. While highly effective, PD-1 blockade can provoke immune-mediated cutaneous adverse events, including eruptive squamous cell carcinomas and keratoacanthomas. These squamoproliferative lesions may reflect a reactive epidermal proliferation triggered by immune activation, potentially through unmasking of subclinical epidermal dysplasia. Histologically, keratoacanthomas and well-differentiated squamous cell carcinomas can be difficult to distinguish, and clinicopathologic correlation is essential for guiding management.

We present a case of a patient receiving pembrolizumab and axitinib for renal cell carcinoma who developed a high burden of eruptive squamoproliferative lesions across multiple anatomic sites. Biopsies were interpreted as either well-differentiated squamous cell carcinoma or keratoacanthoma-type squamous proliferations, underscoring the diagnostic ambiguity inherent to these lesions. Most lesions were successfully managed with intralesional triamcinolone, allowing continuation of immunotherapy without interruption. Two subsequent lesions required surgical excision due to clinical concern for invasive behavior, although no aggressive histologic features were identified.

This case adds to the growing literature supporting conservative dermatologic management of pembrolizumab-associated squamoproliferative lesions. Unlike previous reports describing only a small number of lesions, this case involved a high lesion burden treated with intralesional corticosteroids across multiple sites, with clearly documented technique and follow-up. The case reinforces the clinical importance of correlating histopathologic findings with therapeutic response and demonstrates that, with appropriate monitoring, immunotherapy can often be continued safely while managing cutaneous findings locally.

## Introduction

Cutaneous immune-related adverse events are increasingly encountered in oncology clinics with the widespread use of immune checkpoint inhibitors such as pembrolizumab [1-4]. These reactions range from mild inflammatory eruptions to severe blistering disorders, and in some cases, eruptive squamoproliferative lesions such as keratoacanthomas and squamous cell carcinomas [5-9]. Although these lesions are less common, they present a diagnostic and management challenge due to overlapping clinical and histologic features [7,9-10].

Distinguishing reactive epidermal proliferations from true malignancy is important to avoid unnecessary interruption of potentially life-prolonging therapy [7,9]. The case presented here contributes to the growing recognition of pembrolizumab-associated squamoproliferative eruptions and highlights the use of intralesional corticosteroids as an effective treatment approach that enabled uninterrupted immunotherapy [7,9-10]. Unlike previously published reports, which describe a small number of lesions, this case involved a higher lesion burden treated across multiple sites, with clearly documented technique and follow-up. Dermatologic involvement was essential in guiding management and facilitating continued oncologic care.

## Case presentation

A 65-year-old man with a history of metastatic clear cell renal cell carcinoma, previously treated with bilateral nephrectomy, presented to Dermatology with a new rash involving the upper limbs and back. The eruption began after he had initiated combination systemic therapy with pembrolizumab and axitinib for advanced renal cell carcinoma. He had received seven cycles of both agents at the time of presentation. He reported itching but denied joint pain, muscle aches, lymphadenopathy, or mucosal involvement. There was no personal or family history of skin cancer or inflammatory skin disease. The patient endorsed moderate sun exposure over his life. He had retired from a desk job.

Examination revealed approximately 10 excoriated, pink, scaly papules measuring 2-3 millimetres on the dorsal forearms, shoulders, and lower back. The initial clinical impression by outside providers had been a psoriasiform drug eruption. The patient was advised to apply triamcinolone acetonide 0.1% cream twice daily and was scheduled for follow-up.

**Figure 1:**
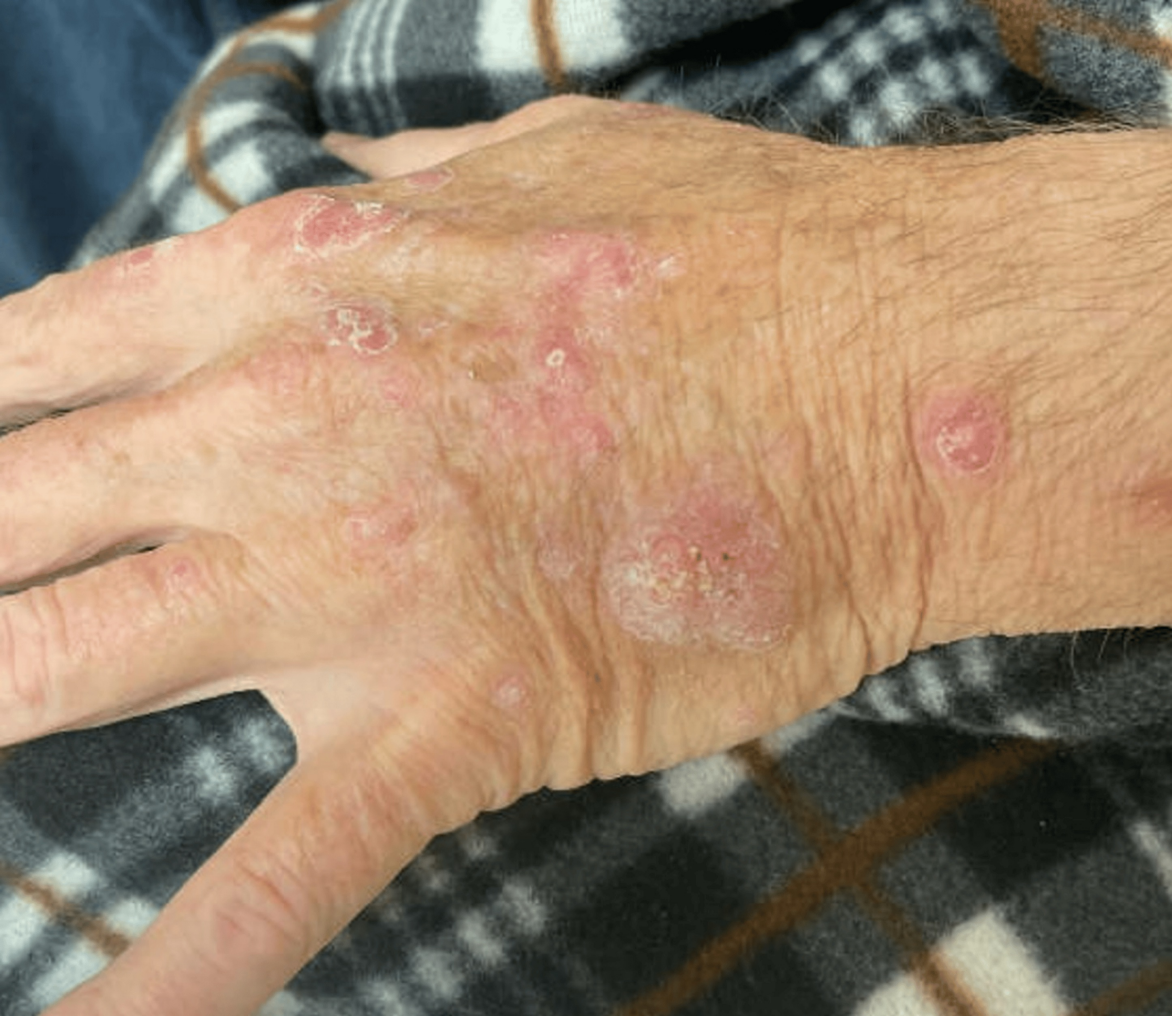
Multiple pink hyperkeratotic papules of the left dorsal hand after initiating pembrolizumab and axitinib

**Figure 2:**
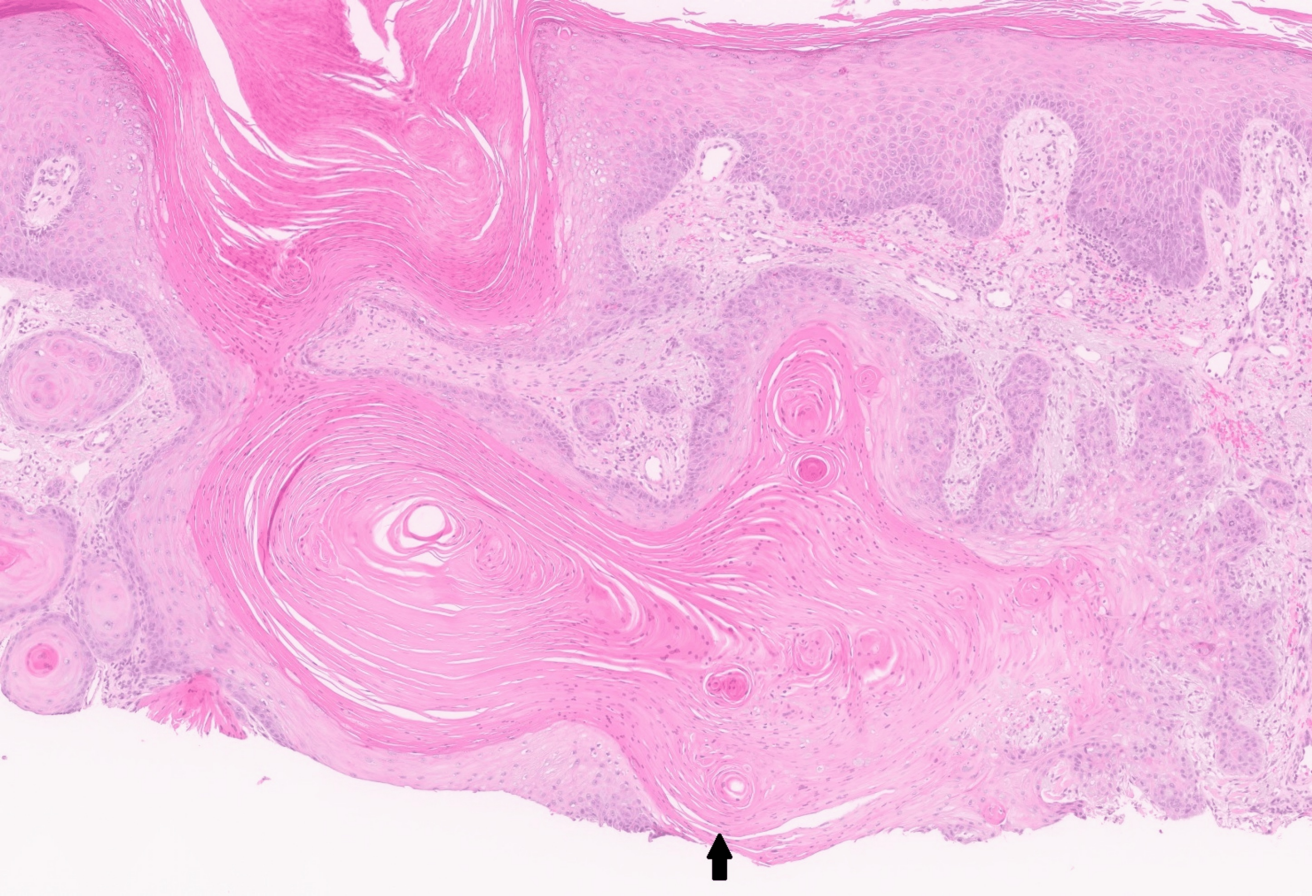
Representative histology at 10x magnification demonstrating keratoacanthoma, deep margin positive, as indicated by the arrow. Key features include keratinizing, eosinophilic keratinocytes.

Three months later, although the itching had improved, the patient noted growth of several lesions on the upper limbs. Scaly papules were noted on the left dorsal hand (Figure 1). Shave biopsies were performed and revealed histopathology most consistent with keratoacanthoma, including crateriform architecture and glassy eosinophilic keratinocytes (Figure 2).

At a subsequent visit, five additional hyperkeratotic papules were identified on both hands. After discussion with the Oncology team, systemic therapy was continued without interruption. Approximately one month following biopsy, the patient underwent two sessions of intralesional corticosteroid injections, spaced three weeks apart. A total of 18 lesions - eight on the left hand, two on the left elbow, and eight on the right hand - were treated with triamcinolone acetonide 10 mg/mL, diluted 1:1 with lidocaine 1%. The total volume injected across all sites was 5.0 mL.

**Figure 3:**
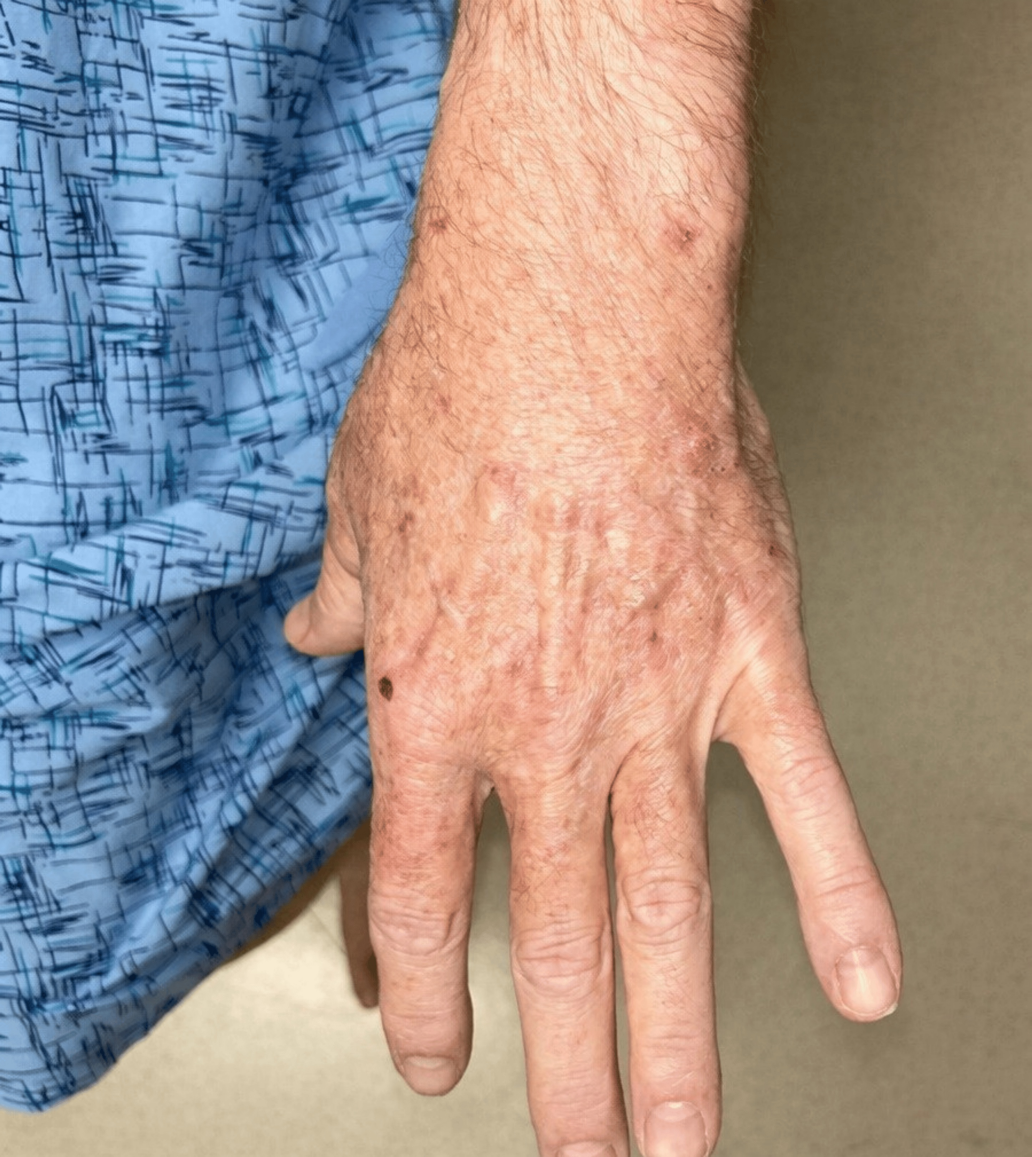
Decrease in size and thickness of keratoacanthomas of the left dorsal hand after treatment with intralesional triamcinolone Note: The papule at the base of the finger is an erosion, not a nevus.

Ten weeks following intralesional triamcinolone treatment, the majority of lesions on the dorsal hands and forearms showed marked improvement in size and thickness (Figure 3). The patient reported no new pruritus or functional limitations in daily activities. Systemic therapy with pembrolizumab and axitinib was continued throughout without interruption.

The patient has continued regular dermatologic surveillance and oncologic care. No recurrence of treated lesions or development of additional eruptive squamoproliferative lesions has been observed during follow-up to date. No cutaneous or systemic adverse events related to treatment have occurred since the resolution of the described lesions.

## Discussion

Pembrolizumab is a programmed cell death protein 1 (PD-1) inhibitor approved for multiple malignancies, including renal cell carcinoma in combination with axitinib [2]. Although highly effective, PD-1 blockade can provoke a wide range of immune-mediated cutaneous adverse events, including bullous pemphigoid, lichenoid eruptions, and squamoproliferative lesions [1,3]. In contrast, axitinib has a relatively limited dermatologic toxicity profile [4].

Eruptive squamous cell carcinomas and keratoacanthomas have been reported in patients receiving pembrolizumab, most frequently on sun-exposed areas of the distal extremities [5-8]. These lesions may reflect a reactive epidermal proliferation triggered by immune system activation [9]. One proposed mechanism involves unmasking of subclinical epidermal dysplasia, leading to a transient hyperproliferative response [9]. This is supported by histologic features such as a lichenoid dermal infiltrate and the clinical pattern of symmetrical lesions in UV-exposed areas [7,9]. In previous cases, keratoacanthoma-like lesions have been reported to respond to topical or intralesional corticosteroids without recurrence or scarring, further supporting an immune-mediated rather than malignant process [7,9-10].

Histologically, keratoacanthomas and squamous cell carcinomas can be difficult to distinguish, and both can appear similar on biopsy [7,10]. In this case, multiple lesions were interpreted as either well-differentiated squamous cell carcinoma or keratoacanthoma-type squamous proliferations, underscoring the diagnostic ambiguity. Clinicopathologic correlation was essential in guiding management.

Several prior case reports have described eruptive keratoacanthomas in patients treated with pembrolizumab [5-8]. In one series, lesions were managed with topical or intralesional corticosteroids, leading to regression without scarring or the need for discontinuation of immunotherapy [7,9-10]. In our case, intralesional triamcinolone was similarly effective for most lesions, allowing continued cancer treatment without interruption. Novel features of this case include the treatment of numerous lesions across multiple anatomic sites and clear documentation of the procedure protocol.

Although both pembrolizumab and axitinib have been associated with squamoproliferative eruptions, pembrolizumab is more frequently implicated in the literature [5-8]. The differential diagnosis may also include hypertrophic lichen planus, lichenoid drug reactions, and well-differentiated squamous cell carcinoma [7,10]. These entities can demonstrate overlapping clinical and histopathologic features, making diagnosis challenging. Biopsy remains important, but histology alone is often insufficient to fully characterize these lesions; clinicopathologic correlation and close follow-up are essential.

This case adds to the growing body of literature supporting the role of conservative dermatologic management in patients with pembrolizumab-associated squamoproliferative lesions. With appropriate monitoring and clinicopathologic evaluation, immunotherapy can often be continued safely while treating the cutaneous findings locally. Unlike previously published reports that described only a small number of lesions, this case involved a high lesion burden treated across multiple anatomic sites using intralesional corticosteroids, with clearly documented technique and follow-up. This case also reinforces the clinical importance of correlating histopathologic findings with therapeutic response in managing these complex lesions. Limitations of our case include a lack of histologic confirmation of the resolution of lesions with treatment and limited follow-up.

## Conclusions

Eruptive keratoacanthomas and squamous proliferative lesions can occur in patients receiving pembrolizumab, and may exhibit overlapping histologic features. Intralesional triamcinolone is an effective treatment for pembrolizumab-associated keratoacanthoma-type lesions, enabling continuation of immunotherapy. Clinicopathologic correlation is essential for guiding the diagnosis and management of cutaneous squamoproliferative eruptions. Dermatologists play a key role in managing immune-related cutaneous adverse events, often enabling the continuation of systemic therapy safely.
